# Care and cost trajectories of asylum seekers in a nurse-led, patient centered, care network in Switzerland

**DOI:** 10.1186/s12913-021-06644-5

**Published:** 2021-07-10

**Authors:** Jacques Spycher, Patrick Bodenmann, Raphaël Bize, Joachim Marti

**Affiliations:** 1 Department of Epidemiology and Health Systems, Center for Primary Care and Public Health (Unisanté), University of Lausanne, Lausanne, Switzerland; 2 Department of Vulnerable Populations and Social Medicine, Center for Primary Care and Public Health (Unisanté), University of Lausanne, Lausanne, Switzerland

**Keywords:** Administrative data, Primary care, Vulnerable populations, Health care costs, Language barriers

## Abstract

**Background:**

Switzerland, with its decentralized health system, has seen the emergence of a variety of care models to meet the complex needs of asylum seekers. A network of public and private providers was designed in the canton Vaud, in which a nurse-led team acts as a first contact point to the health system and provides health checks, preventive care, and health education to this population. In addition, the service plays a case management role for more complex and vulnerable patients. While the network has been examined from a clinical angle, we provide the first descriptive evidence on the care and cost trajectories of asylum seekers in the canton.

**Methods:**

We used routinely collected administrative, patient-level data in a Swiss region responsible for 10% of the asylum seekers in the country. We extracted data on all asylum seekers aged 18 or older who entered the network between 2012 and 2015. The data covered all healthcare costs during the period until they left the network, either because they were granted residence, they left the country, or until 31 December 2018. We estimated random effects regression models for costs and consultations within and outside the network for each month of stay in the network. We investigated language barriers in access to care by stratifying the analysis between patients who spoke one of the official Swiss languages or English and patients who did not speak any of these languages.

**Principal findings:**

We found that both overall health care costs and contacts with the nurse-led team were relatively high during the first year of stay. Asylum seekers then progressively integrated into the regular health system. Individuals who did not speak the language generally had more contacts with the network and fewer contacts outside.

**Conclusions:**

In this exploratory study, we observe a transition from nurse-led specific care with frequent contacts to care in the regular health system. This leads us to generate the hypothesis that a nurse-led, patient-centered care network for asylum seekers can play an important role in providing primary care during the first year after their arrival and can subsequently help them navigate autonomously within the conventional healthcare system.

**Supplementary Information:**

The online version contains supplementary material available at 10.1186/s12913-021-06644-5.

## Introduction

Civil wars, armed conflicts, and poverty have led to an increasing number of individuals moving, often against their will, across borders and seeking asylum in more stable and safer regions [1]. Switzerland has experienced several episodes of increased migration, especially from former Yugoslavia in the 1990s and, more recently, from the Middle East and eastern Africa. Specifically, in 2015, the country saw the arrival of approximatively 40,000 new asylum seekers, mostly from Eritrea, Afghanistan and Syria, a 66% increase from 2014 [2].

The healthcare needs of asylum seekers are complex, because of their very specific profiles in terms of age, socioeconomic status, and culture and given the adversity of their trajectories and experiences [3–5]. In particular, studies have shown a high prevalence of mental health issues in this population, and have highlighted the challenges posed by language barriers in accessing care [1, 6, 7]. Meeting the needs of asylum seekers is challenging for health systems that are often under significant financial pressure and that may face capacity constraints. For many European countries, the recent migration crisis in Europe (2015-2016) posed very specific challenges in terms of providing even basic needs to asylum seekers [8–10], with different policy and organizational responses depending on the resources available, the type of health systems, and political priorities [10–12].

Switzerland is characterized by a strongly decentralized and fragmented health system, with many key decisions in terms of health care planning, financing and organization made at the level of the 26 cantons (i.e., “states”) [13], giving rise to very different responses to the issue of asylum seekers’ care. Several models have emerged, from minimum provision and coordination in some cantons to comprehensive management in others [14, 15]. In the canton Vaud, which is in the French-speaking part of the country and accounts for 10% of the Swiss population, an existing nurse-led, network-based, model called the “RESAMI” (“Réseau santé et migration”, i.e., Network for Migrant Health) has been in place since 2014. This model, which replaced a similar system in place since 1999, emerged from a collaboration between public administrative, and health care stakeholders and private insurers. The model, inspired by the Dutch example [16, 17], is particularly innovative in the country, in terms of care access and coordination, and financing [18]. While previous studies have demonstrated the appropriateness of care delivered [15, 16], an analysis of care trajectories and costs is currently lacking.

In this paper, after describing the RESAMI model, we exploit a large, patient-level, linked dataset to describe the care and cost trajectories of asylum seekers who entered the canton Vaud between 2012 and 2015. We focus the analysis on how access to various care settings – including the RESAMI network, emergency services, and conventional outpatient care – and their associated costs evolve over time. Furthermore, we describe the impact on trajectories of language barriers.

## Background information

In Switzerland, health insurance is mandatory for all residents [14]. Coverage is comprehensive but out-of-pocket payments are typically high because of a high degree of cost-sharing. Insurers are private firms, but they are not allowed to make a profit on mandatory health insurance and must provide coverage to anyone who seeks it. Premiums are not income rated. Low-income individuals can apply for a subsidy, which is allocated according to canton-level rules.

Evidence on the health care use and cost trajectories of asylum seekers is scarce in the country. For instance, some evidence from Basel suggests that asylum seekers have lower mean health care costs than a comparable group of the local population [3]. Studies conducted in Bern show that this population suffers from a variety of somatic symptoms and psychiatric disorders [19] and that a significant percentage is multimorbid and exhibits underlying psychiatric, infectious or chronic medical conditions despite their young age [20]. Evidence from Germany shows higher health care costs for asylum seekers, driven by higher rates of emergency consultations and hospitalizations [21], and finds that restricting access to health care in this population gave rise to higher health care costs overall [22].

Upon entering the country, asylum seekers are directed to one of six federal processing centers. After a first assessment, they are dispatched to various cantons following an allocation rule based on population size (approximatively 10% of asylum seekers are allocated to canton Vaud), available capacity and personal characteristics such as spoken language or the presence of family members. Note that this describes the system during our study period. In March 2019, a new system was implemented (see [18] for details).

**Fig. 1:**
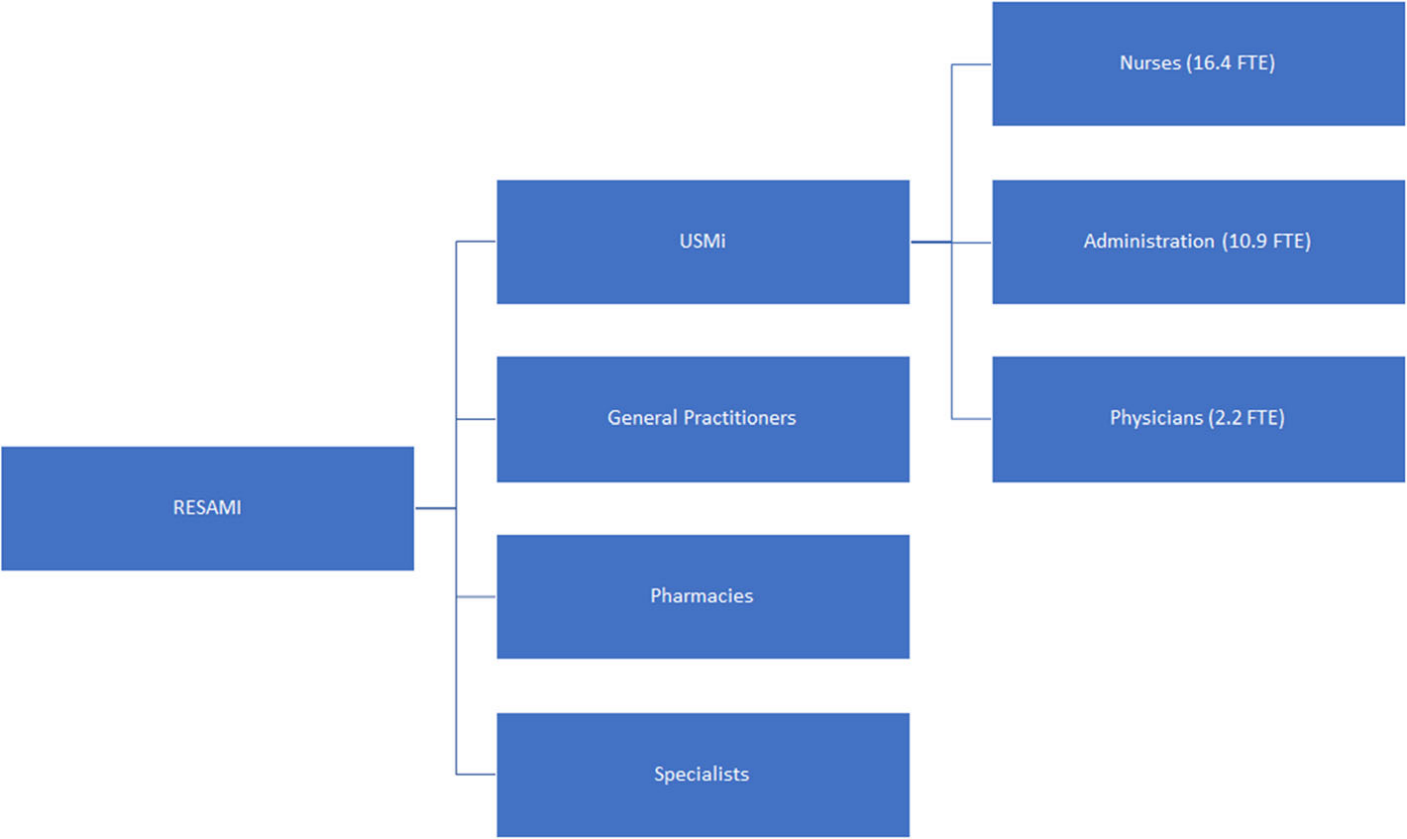
Organization of RESAMI

In 2012, 1460 asylum seekers arrived in the canton Vaud for a population of approximativley 730,000, and the number of arrivals increased to 1951 in 2015 for a population of approximatively 770,000. Housing and social facilities for asylum seekers are under the responsibility of a public institution called EVAM (“Etablissement Vaudois d’Accueil des Migrants”, i.e., Institution for Migrant Reception of Canton Vaud). This institution manages 12 housing facilities across the canton and oversees the registration of arriving asylum seekers. EVAM provides basic information on healthcare to arriving asylum seekers whose administrative data are transmitted to the RESAMI, a network of healthcare providers comprised of the USMi (“Unité de Soins aux Migrants”, i.e., Migrant Care Unit), general practitioners, pharmacies, and specialists. The Migrant Care Unit, which is a single department inside a large university center for primary care and public health (called Unisanté since January 2019), is a nursing unit specialized in the healthcare provision and treatment of asylum seeker populations, with advanced nurse practitioners and interpreters. The service employs a team of nurses (full-time equivalent of 16.4), an administrative team (FTE of 10.9) and physicians (FTE of 2.2) (see Fig. 1).

The mission of the RESAMI is twofold. First, it aims to provide quality healthcare and a first contact point for asylum seekers. Second, it informs this population about the Swiss healthcare system and provides support for their integration into the Swiss population. The Migrant Care Unit provides first line healthcare, immunizations, and information on disease prevention. The other members of the RESAMI support the Migrant Care Unit when more specialized care is needed. Cooperation between RESAMI members is intended to improve continuity of care and reduce administrative costs.

Upon entering the canton, asylum seekers are invited by the Migrant Care Unit to receive a complete health check, complete a vaccination program, and receive information on disease prevention and health promotion. This so called “community health” phase can take up to 1 year to complete. After this initial phase, most individuals should be able to navigate the “conventional” healthcare system, but the Migrant Care Unit is still accessible to patients who request it. However, the service focuses its efforts on those deemed the most vulnerable, based on subjective clinical assessment [23, 24]. For patients with heavy disease burdens who require regular access to multiple specialists, the Migrant Care Unit acts as a case manager.

An important point is that adherence to the RESAMI is not mandatory but is strongly encouraged for the first 12 months of stay. The RESAMI provides specific services aimed at the asylum seeker population, such as specialized nurses and interpreters. Asylum seekers in the canton have the same level of insurance coverage and freedom of access as any permanent resident; the major difference is that premiums and out–of-pocket costs are covered by the canton and not by the individual. The canton buys the same plan, with a CHF 2500 deductible, for all asylum seekers and covers health expenses until the deductible is reached. The health insurance then covers the excess costs. Insurance contracts are randomly dispatched in a pool of private insurers. This system limits financial barriers to health care for asylum seekers, except for some small transportation costs.

## Methods

### Data

We have data on all asylum seekers who entered the canton between 1 January 2012 and 31 December 2015, and were 18 or older at the time of entry. The data cover the period until they leave EVAM and the RESAMI either because they are granted residence, because they leave the country, or until 31 December 2018 (i.e., the end of the data extraction period).

Our data come from three complementary sources, each representing a different institutional partner of the RESAMI network. Administrative data come from EVAM and contain information such as month of arrival (in the canton and in the country), gender, family situation, region of origin (according to the six regions defined by the WHO [25]), spoken language (French, German, Italian, English, or other), legal status, and type of housing (individual or collective). More details about country of origin were not available because of the conditions of anonymity specified by the ethics committee. The second data source comes from the Migrant Care Unit. They gave us access to the data on date, length and type of consultations, referrals to physicians and specialists, and type of interpreter present. The third source is an insurance broker, which gave us information on health expenses and the categories of expenses. Those categories remain at an aggregated level. For instance, we can tell the difference between hospital care and ambulatory care, but we cannot distinguish between general practitioners and specialists. Finally, we have detailed data on drug prescriptions with drug type, quantity, and price.

We linked these data using a unique individual identification code, which was encrypted with the same encryption key by the three external data providers prior to reception of the data. The research team was therefore not able to trace the data back to identifiable individuals. We organized our data in a panel format and defined the time dimension as a count of the number of months of stay since arrival in the canton. The identification code was missing for 11% of the observations in one of the sources (Migrant Care Unit) because other means of patient identification were used internally. After data cleaning, we had observations on 5201 individuals (out of the 5815 eligible) followed during a mean length of stay of 20 months.

### Statistical analyses

We estimate random effects regressions for costs, Migrant Care Unit contacts, contacts outside the Migrant Care Unit, and emergency contacts, controlling for a series of individual-level variables. We define costs as the total monthly invoice amount to which we add an estimate of the monthly Migrant Care Unit cost. The per-consultation cost at the Migrant Care Unit is calculated as their total annual budget divided by the total number of consultations in a year. This is multiplied by the monthly number of consultations for each individual. Migrant Care Unit contacts are the number of contacts with the Migrant Care Unit within a month. Contacts outside the Migrant Care Unit are the monthly number of consultations with an ambulatory care service in the general health system (including hospitals, clinics, general practitioners, and specialists). Emergency contacts are the number of visits to an emergency department in a month.

The main variables of interest are time dummies for each month of stay. We include, as control variables, a gender dummy, a dummy for people older than 50, language dummies, family situation dummies, region of residence (within the canton) dummies, region of origin dummies, a dummy for those in collective housing, a dummy for those under emergency assistance (i.e., basic living assistance provided to asylum seekers who were denied asylum and are waiting to be deported, or to immigrants in illegal situations), and a dummy for those with at least one prescription of psycholeptics and/or psychoanaleptics (mostly antidepressants). We limit the analysis to the first 30 months of stay because of a very small sample size beyond this point. Statistical analyses were performed with STATA the data analysis and statistical software (version 15.1, StataCorp).

Data were encrypted and pseudonymized before reception such that the authors did not have access to any identifying information. All analyses were conducted in accordance with data protection regulations. The study protocol was approved and informed consent was waived by the inter-cantonal ethics committee (“CER-VD: Commission cantonale d’éthique de la recherche sur l’être humain du canton de Vaud”) on 14 June 2018.

## Results

### Descriptive statistics of the sample

Table 1 shows summary statistics for our sample. Twenty-five percent of individuals were women, and 25% were on emergency assistance. Our sample was young with 78% of individuals aged less than 40 years old. Twenty-one percent of the sample spoke French, and more than half (51%) did not speak any of the languages commonly spoken in Switzerland. Close to three quarters were alone (74%). The most represented origins were Africa (49%) and the Eastern Mediterranean (33%). Most of the sample (93%) was housed in collective housing (specifically dedicated to asylum seekers).

**Table 1:** Descriptive statistics of the sample

Variable	N	%
Full sample	5201	100%
Women	1280	25%
Emergency assistance	1312	25%
**Age**		
20 to 24	987	19%
25 to 39	3063	59%
40 to 49	755	15%
50 to 59	237	5%
60 and older	159	3%
**Language**		
Other	2673	51%
French	1087	21%
German, Italian, English	1441	28%
**Family situation**		
Couple	178	3%
Couple with child(ren)	859	17%
Alone	3866	74%
Alone with child(ren)	298	6%
**Origin**		
Stateless	3	< 0.1%
Unknown	82	2%
Africa	2539	49%
Americas	13	< 1%
South East Asia	160	3%
Europe	571	11%
Eastern Mediterranean	1737	33%
West Pacific	96	2%
**Housing**		
Private lease	246	5%
Collective	4854	93%
Individual	100	2%
Hospital	1	< 0.1%
	**Mean**	**Range**
Length of stay (months)	20.0	1-82
**Monthly cost**		
MHI^a^	445.8	0-33,773
Migrant Care Unit	266.0	3.2-2859
Total	711.8	11-33,964
**Monthly contacts**		
Migrant Care Unit	1.01	0-10.9
Outside Migrant Care Unit	0.57	0-4.4
Emergency	0.11	0-2.3

^a^MHI represents mandatory health insurance. These are the costs obtained from invoice data. The first column shows the number of individuals for categorical variables and the mean for continuous variables. The second column shows the proportion for categorical variables and the range for continuous variables

The mean length of stay in the EVAM was 20 months, although there was great variation with a range of 1 to 82 months of stay. The mean monthly health care cost was CHF 711, of which CHF 266 was related to Migrant Care Unit contacts (i.e., not covered by mandatory health insurance, but covered by the cantonal budget) and CHF 445 was related to health care services and prescriptions covered by mandatory health insurance. Some individuals displayed high needs, with monthly costs up to CHF 33,964. On average, individuals had 1.1 contacts with the Migrant Care Unit, 0.6 contacts outside the Migrant Care Unit, and 0.1 emergency contacts in a month.

### Description of care and cost trajectories

**Fig. 2:**
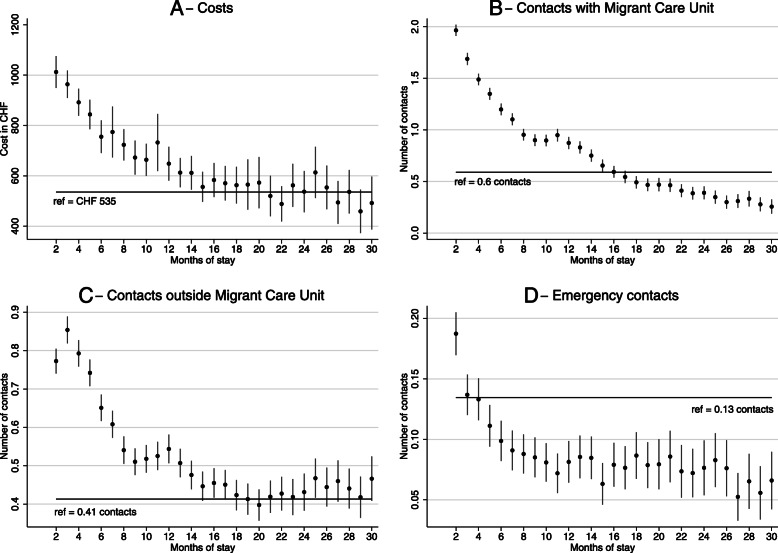
Evolution of monthly costs and monthly contacts during the first 30 months of stay. Results of a random effects regression model with the first month used as a reference, and controls for demographic variables. Symbols indicate coefficients; vertical bars indicate 95% confidence intervals; horizontal lines indicate values for the first month. **A** Evolution of monthly costs. **B**-**D** Evolution of monthly contacts

During their first month of stay, individuals (*N* = 5201) incurred a mean cost of CHF 535, and averages of 0.6 Migrant Care Unit contacts, 0.41 contacts outside the Migrant Care Unit, and 0.13 emergency contacts. Figure 2 shows the evolution of costs and contacts over time obtained from the multivariate regression results. The solid horizontal line shows costs in the first month of stay as the reference category in our regression analyses, and the vertical axis shows monthly predicted costs and contacts with confidence intervals. Detailed regression results on which these figures are based, including the effects of control variables are available in the supplementary materials.

On the left-hand side of Fig. 2a, we see the evolution of cost with duration of stay. Individuals generate higher costs between the second and the twelfth month of stay compared to the first month of stay and later periods. After the first year of stay, there are no significant differences in monthly costs compared to the first month. The relatively small value in the first month of stay might reflect a time lag between entry into the canton and access to care as well as the time to identify health needs.

In panel B, we observe a decreasing trend in Migrant Care Unit contacts over time. High service utilization in the first 15 months of stay was followed by low service utilization during the remainder of the stay. Contacts outside the Migrant Care Unit had a flatter trend overall but were more numerous during the first 16 months of stay. The number of emergency contacts did not differ considerably from the reference throughout the study period, although it remained slightly lower than the reference after the 4th month of stay. There was a crossing point at month 16, when the number of Migrant Care Unit contacts became smaller than the number of contacts outside the Migrant Care Unit. This suggests that individuals transition to “conventional” health care providers after an initial phase of treatment characterized by regular contact with Migrant Care Unit services.

**Fig. 3:**
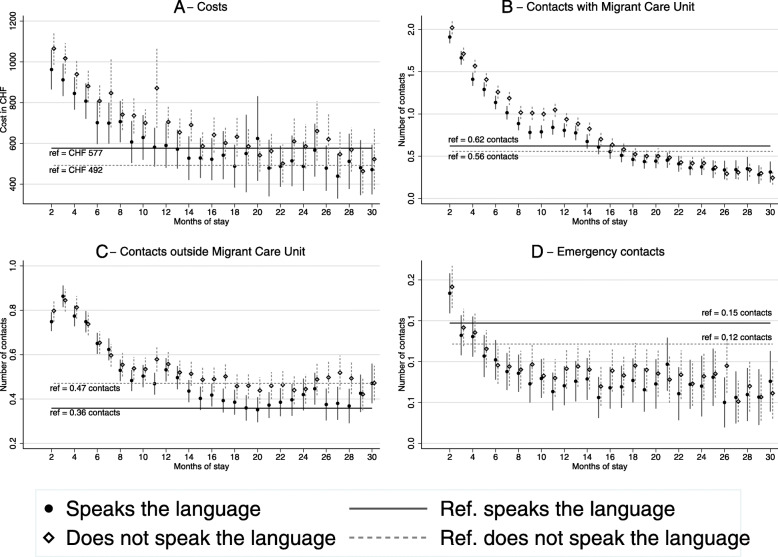
Evolution of monthly costs and monthly contacts stratified by spoken language. Results of a random effects regression model with the first month used as a reference, and controls for demographic variables. Symbols indicate coefficients; vertical bars indicate 95% confidence intervals; horizontal lines indicate values for the first month. “Does not speak the language” refers to individuals who do not speak any of the following languages: French, German, Italian, English

Figure 3 shows the same analysis stratified by spoken language (excluding language as a control in the models). Individuals were classified by their ability to communicate in one of the four languages commonly spoken in Switzerland.. Information on language ability was recorded in the administrative file of each individual, and we were able to distinguish individuals with no competency from those with at least some knowledge of one of the languages (language competency was judged by the administrative staff upon arrival but not based on a specific scale).

Panel A shows that costs were higher for those who spoke an official language than for those who did not. This difference was no longer significant after the first 18 months. Additionally, individuals who spoke an official language had more monthly contacts than individuals who did not, during the 30 months observed. Panel C shows contacts outside the Migrant Care Unit. Here, the difference between groups was no longer observed after the first year of stay. During the first year, individuals who spoke an official language had more monthly contacts between months 8 and 12. Finally, panel D shows that those who spoke an official language had more frequent emergency contacts than those who did not, although the difference was small and not always significant. The number of monthly emergency contacts goes below the reference after the 4th month of stay for both subsamples.

## Discussion

The aim of this paper is to analyze costs associated with a model of care for asylum seekers developed in the canton Vaud in Switzerland. In this model, a nurse-led team acts as a first contact point to the health system and provides – on a voluntary basis - health checks, preventive care, and health education to all asylum seekers in the canton. In addition, the service plays a case management role for more complex and vulnerable patients. A combination of public and private financing also allows asylum seekers to access the conventional health system without any financial barrier.

In an analysis of care and cost trajectories of 5201 asylum seekers who entered the canton Vaud between 2012 and 2015, we found that both costs and Migrant Care Unit contacts were higher during the first year of stay whereas there were fewer contacts within the conventional health care system during the same period. Migrant Care Unit contacts were replaced by contacts outside the Migrant Care Unit after the first year. This result leads us to generate a first hypothesis: The high costs and utilization observed during the first year were caused by the significant health needs of asylum seekers upon arrival. Our second hypothesis is that individuals relied less on the Migrant Care Unit for their health needs after the first year because they were taught to navigate the regular health system. The design of the current study did not allow us to explore potential causal relationships, hence, to formally test these hypotheses would be the subject of future studies.

Individuals who did not speak the languages commonly spoken in Switzerland generally had fewer Migrant Care Unit contacts, suggesting that language barriers might still exist despite the availability of interpreters. On the other hand, we found a similar number of contacts outside the Migrant Care Unit, possibly due to more imperative healthcare needs when individuals receive care from physicians outside the Migrant Care Unit. Individuals who did not speak an official language had lower costs, potentially reflecting language as a barrier to integration and access to care, as evidenced elsewhere [26].

The main strength of this study, beyond describing a particular public-private model in an insurance-based, health system, is the availability of rich person-level data on administrative dimensions, nurse-led activities, and health care service use and costs. These data provide initial insights into health care cost trajectories in a quasi-exhaustive sample of the asylum seeker population of the canton (i.e., 89% of asylum seekers who arrived between 2012 and 2015), which complements existing evidence on the appropriateness of care provided in the network [16]. However, our study has several limitations. While the data are of high quality in several aspects, we did not have access to good diagnostic information on individuals except for broad categories that could be derived from prescription drugs. We lost approximatively 11% of eligible individuals due to data linkage issues. We were not able to identify whether contacts outside USMi took place with a physician who was a member of the RESAMI network. This limitation is somewhat reduced because the costs of visits to a GP are covered by health insurance for both members of the network and those outside of it. Additionally, the timeframe did not cover the peak of arrivals of 2015-2016. Finally, because the RESAMI model is implemented in the whole canton, it is challenging to measure any causal impact of the network on health care use and costs and our approach is therefore purely descriptive. Further studies could for instance focus on a cross-cantonal comparison of health outcomes and costs in the asylum seeker population, exploiting the important heterogeneity of models observed. From a data perspective, our work demonstrates the feasibility of linking administrative, sociodemographic, and health-related data to monitor and evaluate asylum seeker health care services in Switzerland. This is of crucial importance to ensure that this population receives adequate care and that the services are organized efficiently.

One of our main findings is that despite the absence of an obligation to turn to the Migrant Care Unit for primary care, asylum seekers preferentially resort to the unit during their first year in the Vaud canton. Health workers with specific transcultural competencies and a good linkage between EVAM and the Migrant Care Unit may represent strong enough incentives for asylum seekers to follow the recommendation to resort to the Migrant Care Unit first when looking for health care. Additionally, language barriers to accessing health care remain difficult to overcome despite interpreters being part of the health care services that are provided to asylum seekers. Conclusion.

In this exploratory study, we observe a transition from nurse-led specific care with frequent contacts to care in the regular health system. This leads us to generate the hypothesis that a nurse-led, patient-centered, non-binding care network for asylum seekers can play an important role in providing primary care during the first year after their arrival in a new country and help them navigate autonomously within the conventional health care system afterward. Testing this hypothesis will be the work of further research.

## Supplementary Information

**Additional file 1.**

## Data Availability

The data that support the findings of this study are available from the RESAMI network institutions, but restrictions apply to the availability of these data, which were used under license for the current study and therefore are not publicly available. However, data are available from the authors upon reasonable request and with permission of RESAMI network institutions.
